# 5G Infrastructure Network Slicing: E2E Mean Delay Model and Effectiveness Assessment to Reduce Downtimes in Industry 4.0

**DOI:** 10.3390/s22010229

**Published:** 2021-12-29

**Authors:** Lorena Chinchilla-Romero, Jonathan Prados-Garzon, Pablo Ameigeiras, Pablo Muñoz, Juan M. Lopez-Soler

**Affiliations:** 1 Department of Signal Theory, Telematics and Communications, University of Granada, 18014 Granada, Spain; jpg@ugr.es (J.P.-G.); pameigeiras@ugr.es (P.A.); pabloml@ugr.es (P.M.); juanma@ugr.es (J.M.L.-S.); 2 Research Center on Information and Communication Technologies, University of Granada, 18014 Granada, Spain

**Keywords:** infrastructure slicing, network slicing, private networks, 5G, delay, response time, isolation

## Abstract

Fifth Generation (5G) is expected to meet stringent performance network requisites of the Industry 4.0. Moreover, its built-in network slicing capabilities allow for the support of the traffic heterogeneity in Industry 4.0 over the same physical network infrastructure. However, 5G network slicing capabilities might not be enough in terms of degree of isolation for many private 5G networks use cases, such as multi-tenancy in Industry 4.0. In this vein, infrastructure network slicing, which refers to the use of dedicated and well isolated resources for each network slice at every network domain, fits the necessities of those use cases. In this article, we evaluate the effectiveness of infrastructure slicing to provide isolation among production lines (PLs) in an industrial private 5G network. To that end, we develop a queuing theory-based model to estimate the end-to-end (E2E) mean packet delay of the infrastructure slices. Then, we use this model to compare the E2E mean delay for two configurations, i.e., dedicated infrastructure slices with segregated resources for each PL against the use of a single shared infrastructure slice to serve the performance-sensitive traffic from PLs. Also we evaluate the use of Time-Sensitive Networking (TSN) against bare Ethernet to provide layer 2 connectivity among the 5G system components. We use a complete and realistic setup based on experimental and simulation data of the scenario considered. Our results support the effectiveness of infrastructure slicing to provide isolation in performance among the different slices. Then, using dedicated slices with segregated resources for each PL might reduce the number of the production downtimes and associated costs as the malfunctioning of a PL will not affect the network performance perceived by the performance-sensitive traffic from other PLs. Last, our results show that, besides the improvement in performance, TSN technology truly provides full isolation in the transport network compared to standard Ethernet thanks to traffic prioritization, traffic regulation, and bandwidth reservation capabilities.

## 1. Introduction

Fifth Generation (5G) is recognized as a key enabler for Industry 4.0 (the fourth industrial revolution) and its underlying industry digitisation. Smart factories need advanced wireless connectivity to remove the access cabling, which is expensive and cumbersome, prohibits high connection density, and inhibits the mobility of workers and machines. 5G will also enable a myriad of emerging applications to unleash the full potential of the digital transformation of the industry. For instance, wireless-enabled industrial applications include monitoring and controlling cyber-physical systems, industrial Augmented Reality (AR)/Virtual Reality (VR) services, Automated Guided Vehicle (AGVs), and plant monitoring and assessment through massive wireless sensor networks, to name but a few. The heterogeneous and stringent connectivity requirements in latency, connection density, and reliability of these industrial services can be uniquely delivered by 5G to date [1]. According to [2], the service provider addressable 5G-enabled market in the manufacturing industry is foreseen to be USD 132 billion in 2030 with a remarkable compound annual growth rate (CAGR) of 75 percent over 2020–2030, which is concrete evidence that 5G in the industry holds out great promises in terms of connectivity.

5G includes network slicing capabilities to support the traffic heterogeneity expected in the industry. To that end, 5G enables the coexistence of multiple network slices, each tailored for specific services. Although 5G network slicing ensures a certain degree of isolation among network slices, there are use cases that require a more robust level of isolation than the one provided by the traditional network slicing technique. By way of illustration, many 5G industrial use cases require the deployment of several private 5G networks for distinct tenants (multi-tenancy) within the same private venue. To realize these use cases, infrastructure network slicing might be appealing as it offers a higher degree of isolation than 5G network slicing built-in capabilities. Infrastructure network slicing can be regarded as an extension of the notion of network slice to offer a higher degree of isolation, becoming a highly suitable option for multi-tenancy support in private 5G networks. More precisely, an infrastructure network slice is an on-premise slice with dedicated and well isolated resources at every network domain through the use of resource quotas. Another meaningful application of infrastructure networks slicing is the creation of independent and well isolated 5G networks to serve the traffic from different parts of the factory For instance, we can use a dedicated 5G network per production line (PL). In this way, any possible failure affecting one PL will not have an impact on the rest of the factory, thus minimizing the production downtimes and the associated expenditures. Quite costly unplanned downtimes might not be affordable by some industries.

The primary goal of this paper is to evaluate the degree of isolation offered by the infrastructure slicing concept. To that end, we develop a Queuing Theory (QT)-based model to estimate the end-to-end (E2E) mean response time of the infrastructure slices. Specifically, we model a 5G System (5GS) deployed on an infrastructure slice as an open queuing network. The resulting queuing network is solved (its E2E mean delay is estimated) using the Queuing Network Analyzer (QNA) method. Then, we use this model to compare the E2E mean packet delay for two configurations in an industrial scenario. In the first configuration, we consider there is a dedicated infrastructure slice for each PL. In contrast, in the second configuration, a single infrastructure slice serves the traffic generated by all the PLs. The industrial scenario considered for the evaluation is a factory floor with four PLs. In this scenario, we simulate the failure of a PL that results in the generation of non-conformant traffic. From the comparison described above, we can prove the effectiveness of the infrastructure network slicing to provide isolation. In other words, for the assumed scenario, verifying whether infrastructure slicing can avoid the malfunctioning of a PL negatively affects the performance of the rest of the industrial manufacturing processes.

Regarding the experimental setup, we rely on empirical data from the literature and realistic simulations to configure the different input parameters of the proposed analytical model for estimating the E2E mean response time of the infrastructure slices. The resulting complex configuration framework might serve the research community for carrying out, for instance, proofs-of-concept and other evaluation studies. The obtained results support the effectiveness of infrastructure slicing to provide a high degree of isolation in performance among the different slices. From these outcomes, it can be deduced that the number and cost of the production downtimes are reduced as the malfunctioning of a given PL does not affect the others.

Furthermore, besides the two aforementioned configurations considered for the assessment of the E2E mean packet delay, we also compare the performance of two different Transport Network (TN) technologies to realize the midhaul network, namely, standard (bare) Ethernet and asynchronous Time-Sensitive Networking (TSN). The midhaul network interconnects the Next Generation NodeB (gNB)-Distributed Unit (DUs) with the gNB-Central Unit (CUs). The results show that standard Ethernet fails to fully isolate the performance of the different infrastructure slices in the Transport Network (TN). On the contrary, TSN not only ensures the full isolation among the slices but also provides deterministic low-latency.

The contribution of this article is threefold:

(i) We propose an analytical model for estimating the E2E mean response time of the infrastructure network slices.
(ii) Based on the developed model, we provide a delay evaluation study to show the effectiveness of the infrastructure slicing to ensure isolation among PLs in order to minimize the cost of production downtimes.
(iii) Last, but not least, we consider a realistic configuration for an industrial scenario that consists of a factory floor with several PLs. More precisely, we derive the configuration of many parameters from experimental data extracted from the literature (accordingly specified in the corresponding sections). Other parameters have been measured through realistic simulations.

The remainder of the paper is organized as follows: Section 2 provides some background information on infrastructure network slicing and isolation. It also revisits the existing works that address the analytical modeling of the E2E delay of network slices and isolation-related assessment in 5G networks. Section 3 includes the abstract model of the system assumed in this work. Section 4 describes the proposed QT-based model to estimate the E2E delay model of the infrastructure slices in an industrial private 5G network. Section 5 details the methods, the scenario, and specific configurations considered in our performance evaluation study. The obtained results are reported and discussed in Section 6. Last, Section 7 includes the future work and concludes the paper.

## 2. Background and Related Works

This section introduces and motivates the infrastructure network slicing and isolation concepts. Also, it gives an overview of the related works tackling the analytical modeling of the E2E delay and isolation-related evaluations in virtualized mobile networks.

### 2.1. Network Slicing and Isolation

Network slicing concept relies on Software-Defined Networking (SDN) [3,4] and Network Functions Virtualisation (NFV) [5,6] paradigms to enable the creation of several E2E virtual networks, referred to as network slices, each tailored for the necessities of specific services, over a common underlying physical network infrastructure [7]. Network slicing is broadly recognized as one of the most important key enablers of 5G networks [7]. Besides supporting a high heterogeneity of services, network slicing allows for the existence of multi-tenant networks in which over-the-top service providers, mobile network operators, and different vertical industries share the same physical network infrastructure [8,9]. One of the primary requirements and challenges to realizing network slicing is providing isolation among the different network slices. Network slice isolation encompasses several dimensions [10,11]:

- The resources ring-fencing of a slice so as not to negatively impact the proper performance of the rest of the slices.
- The communication capabilities between slices, i.e., not supporting the communication between them if full isolation is required.
- Security capabilities in the sense of protection against deliberate attacks between slices.

This work focuses on the first of the three specified isolation dimensions, paying attention to the performance isolation of slices delimiting their resources. In this way, no matter the load or status of one slice, it will not interfere with other slices’ performance.

3rd Generation Partnership Project (3GPP) 5G standards include built-in capabilities for network slicing support. A 5G network slice is defined as a set of network functions tailored for specific services in terms of performance and functionality. In this vein, 3GPP defines a slicing information model in 3GPP TS 28.541 [12], specifying how to build network slices from network functions to meet specific service requirements. However, it does not address the segregation of resources at the network infrastructure stratum, which is of utmost importance to guarantee performance isolation among Ultra-Reliable and Low Latency Communication (URLLC) slices and enable multi-tenancy support through the allocation of dedicated and well-isolated resources to different tenants running their services atop.

In this work, we adopt the infrastructure network slicing concept defined within the 5G-CLARITY project to enable multi-tenancy in private 5G networks [13,14], with the intention of fixing special attention in isolation, pursuing the ring-fencing of resources. 5G-CLARITY slicing concept allows for the creation of multiple 5GSs on top of a common physical network infrastructure comprising radio access, compute and transport nodes. A 5G-CLARITY slice is a logical partition of the network infrastructure layer that provides an isolated execution environment for specific services or tenants. Each 5G-CLARITY slice comprises a set of dedicated and well-isolated resources from the private infrastructure [13,14]. To that end, 5G-CLARITY system includes a management and orchestration stratum for provisioning infrastructure slices and leverages mechanisms to partition the resources across the different network domains (e.g., Radio Access Network (RAN), Core Network (CN), and TN). Consequently, the resources belonging to an infrastructure network slice are defined per resource domain through the use of resource quotas that delimit the amount of resources for each slice:

- Wireless quota: it refers to the spectrum allocated to each slice in each radio access node. 3GPP 5G standards include functionality to abstract the complexity of non-3GPP wireless technologies (e.g., Wi-Fi and Li-Fi) access points making each appear as a single gNB towards the User Plane Function (UPF). Using non-3GPP technologies leveraging this 5G feature is appealing for enhanced throughput and reliability. Please observe that the specification of the wireless quota depends on the Wireless Access Technology (WAT) (e.g., 5G New Radio (NR), Wi-Fi, and Li-Fi).
- Compute quota: it stands for the computational resources dedicated to each slice in each compute node. It includes physical Central Processing Unit (CPU) cores, RAM, disk, and networking resources.
- Transport quota: it is the set of resources allocated to each slice in the TN. The TN provides connectivity among the 5G components. Typically, these resources might include transmission capacity at a given set of links and buffer space at the corresponding transport nodes’ output ports. A Virtual Local Area Network (VLAN) identifier (tag) can be assigned to each slice in order to differentiate traffic from different slices at layer 2 (L2).

Below are three primary use cases in the context of industrial private 5G networks that call for the highest level of isolation among slices as provided by infrastructure network slicing:

(i) Support of industrial URLLC critical services: URLLC critical services of Industry 4.0 impose the most demanding requirements in industrial networks. The restriction and ring-fencing of resources for their dedication to URLLC services is crucial to guarantee the stringent requisites demanded by these kinds of services and applications.
(ii) Network performance isolation of the Operational Technology (OT) domain components: The division/segmentation of an industrial network into well-isolated parts for supporting the operation of disjoint OT components becomes essential to limit the scope of a malfunctioning, thus reducing production downtimes and associated expenditures.
(iii) Multi-tenancy support: Part of the success of private 5G networks will be the ability to allow the provision of communication services from different customers (tenants) with such an isolation level that guarantees the agreed performance and management capabilities. Several use cases requiring multi-tenancy support have been proposed in the literature [15].

### 2.2. Analytical Performance Models for Network Slicing

Some works have proposed analytical E2E delay models for virtualized wireless networks and network slices [16,17,18,19,20,21,22,23,24,25,26,27]. Although there are also works providing performance models for a specific network domain (e.g., RAN, TN, and CN) [28,29,30,31] or component (e.g., gNB and UPF) [32,33], here we will only review E2E delay models, i.e., those considering every network domain. Analytical models are crucial to assist autonomous solutions for the management and operation of the network and to perform offline network performance assessments and optimization. On the one hand, analytical models are essential to proactively compute the configuration and estimate the resources to be allocated according to the expected workload in the near future [34,35]. Also, they serve to ensure the cohesion and satisfiability of the configurations applied to the different network and infrastructure domains. In this regard, the authors in [36] rely on analytical performance models to develop a solution that guarantees smooth communications for E2E service delivery when there is a wide variety of Quality of Service (QoS) classes in each network domain. It shall be noted that alternative approaches such as real-time delay sensing [37] can complement analytical models in many use cases through reactive solutions, i.e., real-time performance monitoring and actuation in case of any performance requirement violation. On the other hand, analytical models serve to fast and effectively verify, for example, whether a network architecture and built-in features meet the target performance requirements. If not, an architectural redesign, optimizations, and new capabilities can be proposed for the network.

Table 1 includes a survey on the research literature addressing the analytical modeling of the E2E delay of virtualized mobile networks and network slices. There are three primary mathematical frameworks used in the literature to develop analytical E2E delay models, namely, QT [38], Deterministic Network Calculus (DNC) [39], and Stochastic Network Calculus (SNC) [40]. These three frameworks model the whole system as a network of queuing facilities, each representing a shared resource (e.g., link capacity) in the system. Broadly speaking, QT mainly addresses queuing systems with renewal arrival and service processes and aims to provide the E2E mean delay. On the other hand, DNC relies on alternative algebras (e.g., min-plus and max-plus) and inequalities to derive the worst-case E2E delay. Last, in contrast to DNC, SNC leverages the stochastic nature of the arrival and service processes to estimate non-asymptotic statistical delay bounds of the form P[d>D]≤ϵ [41], i.e., the probability that the delay of the system *d* be greater than a given delay threshold *D* is bounded by the value of ϵ. Table 1 indicates the mathematical framework used by each revised reference.

Overall, the proposed models in the literature either do not address the specificities of the RAN, such as the co-channel interference, or the features of the URLLC traffic and the respective RAN setup to serve it, which ultimately translate into the radio channel capacity degradation. Similarly, they do not capture traffic prioritization at the TN domain. Deterministic network technologies like TSN are firm candidates to enable the conveyance of the URLLC traffic while allowing for its coexistence with massive machine type and enhanced Mobile Broadband (eMBB) communications over a common TN infrastructure. In this regard, traffic prioritization, which is a key feature of deterministic transport technologies, plays a crucial role in isolating URLLC traffic. Also, the models are tested under simplified or unrealistic setups for industrial private 5G networks. Last, focusing on the QT-based E2E delay models, most of them lack of generality as they assume exponential packet inter-arrival times and exponential (M/M/1-based models) or deterministic (M/D/1-based models) service times with a single server. In [16], the authors consider arbitrary arrival and service processes but still a single server facility at every queue (G/G/1-based model). Although a single server assumption might apply to model the packet transmission in the wired network devices links (e.g., TN links), it does not allow for other potential bottlenecks, such as Virtual Network Functions (VNFs) with several CPU cores/threads processing packets in parallel or the radio interface transmitting several packets simultaneously through orthogonal sets of Physical Resource Blocks (PRBs).

In this work, we cover the gaps mentioned above. To that end, we propose a QT-based model to estimate the E2E mean packet delay of the network slices. More precisely, the network slice is modeled as an open network of G/G/m queues, and the E2E mean delay is estimated using the QNA method proposed by Whitt [42]. The model developed in this work can be regarded as an extension of the one proposed in [26]. In [26], the authors propose a G/G/m-based mean delay model for Softwarized Network Services (SNSs) focusing on the computing domain (e.g., virtualized mobile network cores) and experimentally validate its accuracy. In particular, they report their model achieves less than half of the error in terms of accuracy compared to M/M/m-based models. This is reasonable considering that the QNA method is consistent with the Jackson network theory [42]. A simulation-based validation is also provided in [43]. Other works support the usefulness and accuracy of the same modeling approach for the SNSs resource sizing. Specifically, it is used for SNSs’ planning [31] and Dynamic Resource Provisioning (DRP) in [34,35,44]. Here, we leverage the modeling approach in [26] to develop a generic E2E mean delay model of 5G slices that captures the behavior and features of the RAN and TN network domains. The resulting model is quite general while preserving the simplicity and exhibiting low computational complexity (please refer to the execution times measurements reported in [43]). The model is primarily intended to carry out performance evaluations of 5G network slices as those included in 3GPP technical reports (TRs) (e.g., 3GPP TR 38.802 [45]) or the one included in this work. Furthermore, this work might serve as a basis to extend other works proposing network calculus-based models through reproducing the methods followed in this work for capturing the behavior of many 5G network features.

### 2.3. Network Slicing Isolation Assessment

Isolation is still a challenging requisite to be wholly met in today’s networks. Several works have addressed the degree of isolation offered by network slicing for specific network domains [28,46,47,48,49,50,51]. In [46], two resource allocation methods for isolation in the RAN are presented. They guarantee resource isolation by limiting the maximum allocated resources blocks to each slice and implementing slight modifications of the ordinary packet scheduling algorithms. Their results show that the performance achieved by these methods is improved, especially in high-resource utilization environments. The work in [47] addresses the isolation problem between slices also in the RAN. The authors demonstrate how isolation can be achieved in dynamic network slicing using an appropriate Connection Admission Control (CAC) mechanism. In [51], the authors propose a novel control framework for stochastic optimization based on the Lyapunov drift-plus-penalty method for a wireless system with a time-varying number of users. This method enables the system to minimize power, maintain slice isolation, and provide reliable and low latency communications for the slices that require these requisites. The authors in [28] propose a novel resource slicing scheme focusing on the performance isolation of network slices. To that end, they developed a continuous-time Markov chain to estimate the performance metrics, such as data rate, of the RAN. In contrast to our work that target industrial URLLC services, this work is centered around bandwidth-greedy services. Their results suggest that the proposed approach might double the data rate compared to the complete static segregation of resources. Nonetheless, throughput gains are not the main objective for critical industrial services. Thus, resource sharing might not be justified for critical services as it can compromise performance isolation and hinder the proper operation of them. Regarding the transport domain, in [48] the authors develop a control plane architecture for TSN networks able to support network slicing and show how to preserve slice isolation over a TSN-based TN. With respect to the computing domain, in [52] the authors address the optimal allocation of a slice in 5G core networks by tackling intra-slice function isolation and guaranteeing the E2E delay for a slice.

To the best of our knowledge, up to date, very few works carry out an E2E isolation evaluation. The authors in [49] develop a network slicing approach suitable for the deployment in current SDN and NFV enabled communication infrastructures. The approach is verified by empirical performance evaluation using a physical testing setup that showcases slice isolation even during partial overloads. In [50], the authors present a prototypical realization of E2E network slicing considering radio access and core networks based on NFV and SDN as key technologies. They also provide an empirical evaluation of the proposed E2E network slicing solution based on real-world traffic patterns (e.g., smart grids, intelligent transport, and best-effort (BE)).

## 3. System Model

**Figure 1:**
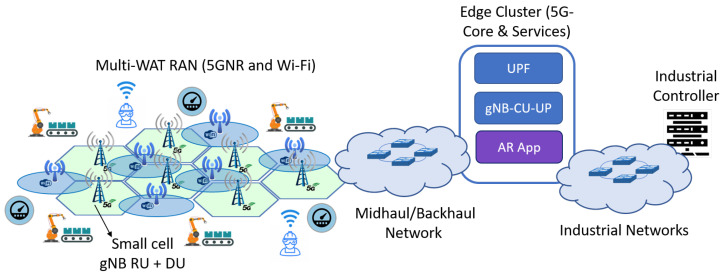
System model: Private industrial 5G network with multi-WAT RAN and the 5G Core deployed on the edge cluster.

This section describes the abstract model of the services and 5GS deployed on an infrastructure slice in an industrial factory floor, together with the main assumptions considered. Broadly, a 5GS provides the OT devices of a factory floor with radio connectivity. Figure 1 sketches a high-level view of the scenario under study in this work.

The factory floor with dimensions W×L m${}^{2}$ includes $N_{PL}$ PLs. Each PL is composed by $N_{URLLC}$ devices to automate the manufacturing process. The network traffic generated by monitoring and controlling these devices has deterministic low-latency requirements, i.e., URLLC traffic. For example, motion control is a typical industrial service in which an industrial controller communicates with remote sensors and actuators to control the motion of industrial machinery. This service has hard real-time requirements, i.e., cycle times and latency are highly critical, to within milliseconds or even microseconds [53].

Dedicated infrastructure network slices are deployed for specific services or set of devices (e.g., sensors and actuators from a given PL). There might be one or several infrastructure network slices to serve the URLLC traffic generated to control and monitor the PLs. A given infrastructure slice might be dedicated for serving the traffic of one PL or shared between several PLs. Each infrastructure slice has segregated resources for every network domain (e.g., RAN, TN, and computing domain), i.e., an infrastructure network slice consists of a set of dedicated and well isolated computational (e.g., physical CPU cores, Random Access Memory (RAM), and disk), transport (e.g., buffer space, and link capacity at every switch egress port) and radio (e.g., buffer space at the radio interface, and bandwidth) resources.

Multi-WAT combining 5G NR and Wi-Fi technologies is considered. We assume Wi-Fi technology does not include deterministic low-latency support, thus it only serves eMBB traffic. In contrast, 5G NR can serve any type of traffic, though in our setup (see Section 5) we consider it only serves URLLC traffic. Although we consider dedicated resources for each infrastructure slice, the eMBB traffic might degrade the performance of URLLC traffic depending on the specific configuration. For instance, when there is no prioritization or resource reservation in the TN, the eMBB traffic will compete for the transmission capacity with URLLC one at some links.

The model description of each of the network domains (computing, RAN, and TN domains) that conform the E2E network layout is included in the subsections below (Section 3.1, Section 3.2 and Section 3.3, respectively).

### 3.1. Computing Domain

The computing domain comprises the compute nodes to host the set of VNFs. Here, we consider the same configuration as that considered in [26,33] for the VNFs of the 5GS. The 5G VNFs (e.g., gNB-CU and UPF) instances have one or several dedicated CPU cores (CPU pinning) in the Physical Machines (PMs) or servers. There is a processing thread per dedicated CPU core allocated to allow for the parallel processing of the packets at the corresponding VNF. There are also CPU physical cores dedicated to the virtualization container housekeeping. We assume software-based with run-to-completion (RTC) pipeline for the VNFs, i.e., all the processing tasks to process each packet are executed at once, followed by the processing of the next packet picked for processing (FCFS discipline). RTC approach for packet processing is highly suitable for the scenario due to the following reasons [33]:

(i) Processing of packets, for instance, in gNB-CU and UPF instances, from each 5G stream is quite independent from other streams. Then, there is no need to divide processing into smaller pieces to spread it across cores.
(ii) RTC mode minimizes the context switchings and maximizes the cache hit rate, which results in a lower packet processing delay.

### 3.2. Radio Access Network Domain

For the User Plane (UP) of the RAN, we consider the baseband functions are split into three components, namely, gNB-CU, gNB-DU, and gNB-Radio Unit (RU). We assume the splitting options #2 and #7 for the F1 (interconnecting gNB-CU and gNB-DU instances) and Fx (interconnecting gNB-DU and gNB-RU instances) [54] interfaces, respectively. In this way, the gNB-CU is in charge of the per packet processing associated with the Radio Resource Control (RRC), Service Data Adaptation Protocol (SDAP), and Packet Data Convergence Protocol (PDCP) protocols. The operation considered for the virtualized gNB-CU is the same as the virtualized UPF’s implementation described in [33] and compatible with the one assumed in [29] for the Cloud RAN’s Baseband Unit (BBU) pool. Thus, the processing of these upper layers is the main potential bottleneck of the gNB-CU. On the other hand, the gNB-DU is responsible for the Radio Link Control (RLC), Medium Access Control (MAC), and part of the physical layer (e.g., equalization and Multiple-Input Multiple-Output (MIMO) precoding). In contrast to the gNB-CU whose packet service time only depends on the workload, the gNB-DU packet processing rate also depends on the carrier bandwidth and Modulation and Coding Scheme (MCS) index [55,56,57]. Last, the gNB-RU realizes the Fast Fourier Transform (FFT)/Inverse Fast Fourier Transform (IFFT), resource mapping and Radio Frequency (RF) functionalities. The packet service time of the gNB-RU depends on the carrier bandwidth and the virtualization layer when the function is virtualized [54,55,56].

There might be multiple gNB instances through the coverage area. The available bandwidth, denoted as BW, is split into *N* channels of $BW_{c}=BW/N$ bandwidth. Several channels are allocated to each gNB instance. A given channel might be shared by multiple gNB instances, resulting in co-channel interference. The attainable data rate for a URLLC device *j* at the radio interface is a function of its allocated bandwidth $BW_{j}$, its perceived Signal-to-Interference-plus-Noise Ratio (SINR) $SINR_{j}$, the packet size and the block length. For low latency applications, there is always a probability of packet drop due to noise. In addition, data must be encoded at a rate significantly lower than that given by the Shannon’s capacity formula in order to get a higher reliability [58,59]. The authors in [59], based on [58], derives the following performance model for the User Equipment (UE) achievable rate:

(1) $$R_{j}=BW_{j}\cdot \left(log_{2}\left(1+SINR_{j}\right)-\sqrt{\frac{C_{j}}{n}}\cdot Q^{-1}\left(\epsilon \right)\cdot log_{2}\left(e\right)\right)$$

where *n* is the blocklength for a given duration τ of the time slot. For instance, a resource block in a Long-Term Evolution (LTE) system contains 84 symbols and lasts 0.5 ms [60]. 5GS allows for the use of flexible numerology which can be translated into configurable values of the time slot duration τ. $Q^{-1}\left(\cdot \right)$ is the inverse of the Gaussian Q function. ϵ is the transmission error probability. $log_{2}\left(e\right)$ refers to the logarithm in base 2 of number *e*. $C_{j}$ is the channel dispersion of the UE *j*, which is given by:

(2) $$C_{j}=1-\frac{1}{{\left(1+SINR_{j}\right)}^{2}}$$

Observe that (1) adds a correction factor to the Shannon’s capacity formula in order to consider the specific physical layer behaviour for URLLCs with small packet sizes, as previously mentioned.

Last, as in [32], we assume that packets are transmitted without errors. In other words, there are no Hybrid Automatic Repeat Request (HARQ) retransmissions.

### 3.3. Transport Network Domain

The 5G components are interconnected through the Transport Network (TN). Here, we consider an asynchronous bridged network (traditional Ethernet or asynchronous TSN networks) for realizing the TN segments shown in Figure 1 (e.g., midhaul and backhaul). On the one hand, traditional Ethernet does not include traffic differentiation capabilities. Then, all types of traffic receive the same treatment. This technology is affordable and easy to configure, but it is hard to support deterministic QoS in these networks [30]. What is more, the computation of the E2E worst-case delay is an nondeterministic polynomial time (NP)-hard problem [61]. On the other hand, asynchronous TSN is more complex to configure [62,63], but provides deterministic QoS through per-link traffic regulation and traffic prioritization [30]. Asynchronous TSN is suitable to serve non-periodic deterministic traffic patterns and enables its coexistence with the best effort one [64]. Thus, it is a perfect candidate to realize the 5G TNs as the traffic types mentioned above are expected to be dominant there [65]. In asynchronous TSN, the transmission of the frames at each link is handled by an Asynchronous Traffic Shaper (ATS). Each ATS has several priority queues to apply strict traffic prioritization [66,67]. Eight priority levels are considered by default in standards [68]. Also, each ATS has a maximum number of shaped buffers for carrying out per-flow traffic regulation. The maximum number of these buffers might limit the number of implementable priority levels [66,67]. We refer the interested reader to [65,66,67,68] for further details on the ATS operation.

The queue to place each frame at a given output port of a TSN switch in the TN is taken according to the Priority Code Point (PCP) of the VLAN tag. We assume there is a mechanism in charge of doing the mapping of 5G streams onto PCPs according to some criteria.

## 4. E2E Mean Delay Model

This section includes the analytical performance model employed for estimating the E2E mean response time of the network slices. A network slice comprises several components (e.g., UPF, gNB-CU, gNB-DU, gNB-RU, and TN bridge) at the data plane. In turn, every component might have several instances, each with multiple resources (e.g., CPU, RAM, disk, and link capacity) supporting the operation of the data plane. We model a network slice as an open queuing network. Each queue in the network models a Primary Resource Access Delay Contribution (PRADC), i.e., the queuing time involved to access a resource associated with a given component instance (for instance, the CPU time at a given UPF instance) supporting the data plane operation that has a non-negligible dependency on the workload. PRADCs are related to the potential bottlenecks of the system. That is, those resources that can potentially have the highest utilization in the system and become the primary source of delay. By way of illustration, the switching fabric of the networking devices, such as bridges, is typically designed to operate at the line rate, and the associated processing is almost constant with the traffic load. Consequently, the packet transmission at the links is usually the PRADC for the networking devices instances in the TN, while the switching packet processing delay can be regarded as constant.

**Figure 2:**
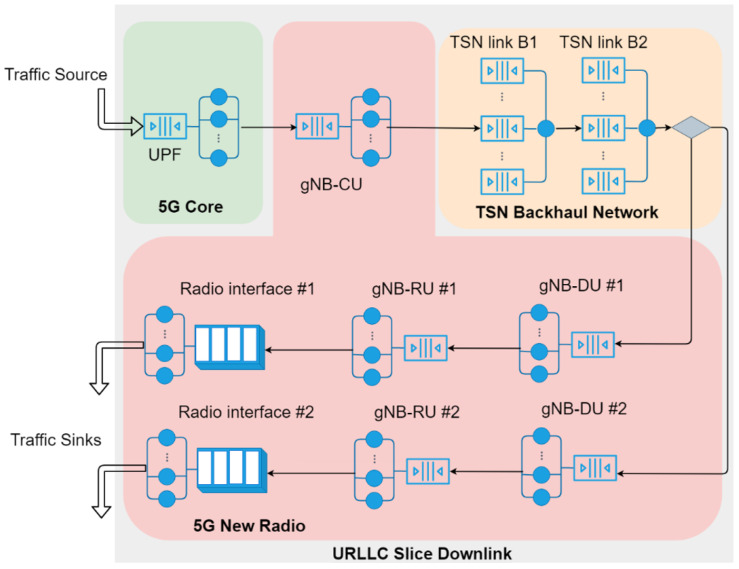
Example of queuing network to model the downlink of a network slice.

Figure 2 shows an example of the queuing model for the downlink of a slice. For simplicity, the figure only depicts the PRADCs, but not the constant delay components, e.g., propagation delay at every link. There is only one instance for each VNF (e.g., UPF and gNB-CU) and two small cells encompassing gNB-DU and gNB-RU functionalities. We assume 5GS components execute CPU-intensive tasks for the packet processing, being the CPU time the only PRADC of the 5GS components instances. Then, the queuing servers of each 5GS component instance in Figure 2 stand for physical CPU cores and the respective processes or threads running the tasks associated with each packet processing in parallel. For example, the service time of every queuing server at the UPF represents the processing time required by the CPU core/thread to run the packet processing task, which is ultimately given by the total number of task instructions to be executed and the processor computing power. Exceptionally, besides CPU processing, the packet transmission at the radio interface is considered another PRADC in the gNB-RU instances. The queuing servers at the radio interface correspond to PRBs and the service time is the time slot duration, which is given by the configured numerology. For the TN segments, the example considers asynchronous TSN as L2 technology. There is a PRADC at each TSN bridge output port related to the frames handling and transmission at a given TN link. Each TSN bridge port is modeled as a non-preemptive multi-priority queuing node, where the server represents the link packet transmission process, whose service time is given by the nominal transmission capacity of the link. The only external packet arrival process to the slice downlink is at the UPF, and the packets leave the queuing network right after they are transmitted through the radio interface.

To solve the resulting network of queues modeling the downlink of the slices, i.e., to estimate the E2E mean delay, we rely on the queuing network analyzer (QNA) method proposed in [42]. This method can be regarded as an extension of the methodology to solve Jackson’s open networks, which consists of M/M/c queuing nodes, to general open networks composed of G/G/c queuing nodes. The most important feature of this method is that it provides approximations to efficiently compute the first and second-order moments of the internal arrival processes at every queue. In [26], this methodology has been experimentally validated to estimate the mean response time of softwarized network services.

Please note that, for generality, we use the indexes *k* and *i*, which represent integer numbers, in the subsequent analysis to differentiate the queues in the queuing network. Let us recall that a queue stands for a PRADC of a given component instance. Please note that the mapping of queues onto indexes (queue-to-index assignment) might be arbitrary, though it has to remain the same for all the computations. The primary notation used through this section is defined in Table 2.

### 4.1. Network Slice End-to-End Mean Response Time

The E2E mean response time *T* of a network slice in the downlink direction can be estimated by adding up the PRADCs, each associated with a given resource in a network component instance, and the constant delay contributions:

(3) $$T=\Phi +\sum_{k=1}^{K}T_{k}\cdot V_{k}$$

where

- Φ stands for the constant delays in the system, i.e., those delay components that do not depend on traffic load (e.g., propagation delays) or those whose dependency on the traffic load is negligible (e.g., switching fabric processing time of the physical L2 bridges or RAM accesses in VNFs when they execute CPU-intensive tasks).
- $T_{k}$ is the mean sojourn time of the queue *k*. As previously mentioned, a queue *k* is associated with a given data plane component or functionality (e.g., UPF, gNB-CU, gNB-DU, gNB-RU, and TN bridge), an instance of the respective component, and a resource within that instance. There is no pre-established rule to perform the queues to numerical indexes mapping, though this assignment shall remain the same for all the computations.
- $V_{k}$ denotes the visit ratio of the queue *k* (a specific resource), i.e., the average number of times a packet or the respective processing task visits the queue *k* since it enters until it leaves the network slice. For instance, a VNF packet processing could be modeled as three queues related to the CPU, RAM, and disk resources, each accessed a given number of times on average to run the packet processing task.

Next, we will specify the mean delay $T_{k}$ computation for each individual queue or PRADC *k*.

### 4.2. Mean Sojourn Time per Queue Computation

The PRADCs might be modeled as G/G/m queues. That is, a queuing facility with general distributions for both the packet inter-arrival and service times and an infinite FCFS queue. The mean response time of this queuing node can be estimated using the following heavy traffic approximation [38]:

(4) $$T_{GGm}=0.5\cdot \left(c_{ak}^{2}+c_{sk}^{2}\right)\cdot \frac{C\left(m_{k},\frac{\lambda_{k}}{\mu_{k}}\right)}{m_{k}\mu_{k}-\lambda_{k}}+\frac{1}{\mu_{k}}$$

where $\lambda_{k}$ and $c_{ak}^{2}$ are the aggregated arrival rate and the SCV of the inter-arrival packet times at queue *k*, respectively. In other words, the first and second order moments of the internal arrival process at queue *k*. Regarding the service process characterization, $\mu_{k}$, $c_{sk}^{2}$, and $m_{k}$ denote the mean service rate, SCV of the service time, and the number of servers at queue *k*, respectively. Last, C(m,ρ) is the Erlang’s C formula for a queuing node with *m* servers and utilization ρ, which is given by:

(5) $$C\left(m,\rho \right)=\frac{\left(\frac{{\left(m\cdot \rho \right)}^{m}}{m!}\right)\cdot \left(\frac{1}{1-\rho }\right)}{\sum_{n=0}^{m-1}\frac{{\left(m\cdot \rho \right)}^{n}}{n!}+\left(\frac{{\left(m\cdot \rho \right)}^{m}}{m!}\right)\cdot \left(\frac{1}{1-\rho }\right)}$$

To model resources including traffic prioritization, for instance, the packet transmission at the asynchronous TSN-based bridges’ output ports includes this feature, we consider the use of non-preemptive multi-priority M/G/1 queues in the resulting queuing network. This queuing node comprises a single server with *P* FCFS priority queues or priority classes. The packets are served from these queues according to a non-preemptive strict priority scheduling. That is, the packets with higher priority are served before packets with lower priority. However, the service process of any packet is not interrupted until it is completed, even though a packet with higher priority arrives meanwhile. Each priority class p∈[1,P] might have a different service process characterized by the mean service rate $\mu_{k}^{\left(p\right)}$ and SCV of the service times $c_{sk}^{2(p)}$. It is assumed that priority class *P* corresponds to the lowest priority. The mean response time experienced by a packet with priority class *p* at this queuing node is given by:

(6) $$T_{NPMP-MG1}^{\left(p\right)}=\left\{\begin{array}{c} \frac{\sum_{r=1}^{P}\lambda_{k}^{\left(r\right)}\cdot \left[\left(c_{sk}^{2(r)}+2\cdot \mu_{k}^{\left(r\right)}-1\right)/{\left(\mu_{k}^{\left(r\right)}\right)}^{2}\right]}{2\cdot \left(1-\rho_{k}^{\left(1\right)}\right)}+\frac{1}{\mu_{k}^{\left(p\right)}}\;if\;\;p=1\\ \frac{\sum_{r=1}^{P}\lambda_{k}^{\left(r\right)}\cdot \left[\left(c_{sk}^{2(r)}+2\cdot \mu_{k}^{\left(r\right)}-1\right)/{\left(\mu_{k}^{\left(r\right)}\right)}^{2}\right]}{2\cdot \mu_{k}^{2}\cdot \left(1-\sum_{r=1}^{p-1}\rho_{k}^{\left(r\right)}\right)\cdot \left(1-\sum_{r=1}^{p}\rho_{k}^{\left(r\right)}\right)}+\frac{1}{\mu_{k}^{\left(p\right)}}\;if\;\;1<p\leq P \end{array}\right.$$

where $\lambda_{k}^{\left(p\right)}$ and $\rho_{k}^{\left(p\right)}=\lambda_{k}^{\left(p\right)}/\mu_{k}^{\left(p\right)}$ are the aggregated mean arrival rate and utilization at priority level *p* of the non-preemptive multi-priority queue *k*.

In a nutshell, we use the G/G/m queue to model a resource that do not support traffic prioritization (i.e., its number of priority levels $P_{k}$ equals one), whereas non-preemptive multi-priority M/G/1 queuing model is employed to estimate the mean response at the priority class $p\in [1,P_{k}]$ for resources supporting traffic prioritization:

(7) $$T_{k}=\left\{\begin{array}{cc} T_{GGm}\left(\lambda_{k},c_{ak}^{2},\mu_{k},c_{sk}^{2},m_{k}\right) & \;if\;\;P_{k}=1\\ T_{NPMP-MG1}^{\left(p\right)}\left(\lambda_{k}^{\left(p\right)}\forall p\in \left[1,P\right],\mu_{k}^{\left(p\right)}\forall p\in \left[1,P\right],c_{sk}^{2(p)}\forall p\in \left[1,P\right]\right) & \;if\;\;P_{k}>1 \end{array}\right.$$

In the following subsections, we describe the estimation of the first and second order moments for both the aggregated arrival and service processes of every queue. Observe that these moments are the primary input parameters to estimate the mean response time per queue in the expressions (4)–(7) introduced above. On the one hand, the QNA method includes approximations to efficiently estimate the aggregated packet arrival rate and SCV of the interarrival packet times at each queue. On the other hand, we rely on the combined use of simulation, experimentation, and analysis to obtain the service processes characteristics of the main PRADCs. It is worth noting that many features of the system behavior considered in this work and presented in Section 3, especially for the 5GS operation, can be captured through the service-related input parameters (i.e., mean and SCV of the service times).

### 4.3. First and Second Order Moments Computation of the Internal Arrival Processes

First, similar to the Jackson’s method to solve open network of M/M/m queues, we calculate the aggregated arrival rate for each queuing facility. Let $\lambda_{k}$ denote the total arrival rate to queue *k*. As in the case of Jackson’s networks, we can compute $\lambda_{k},\;\forall \;\left\{k\in N|1\leq k\leq K\right\}$ by solving the following set of linear flow balance equations:

(8) $$\lambda_{k}=\lambda_{0k}+\sum_{i=1}^{K}\lambda_{i}\nu_{i}p_{ik}$$

To estimate the SCV $c_{ak}^{2}$ of the aggregated arrival process to each queuing node *k*, QNA relies on approximations to derive the following set of linear equations [42]:

(9) $$c_{ak}^{2}=a_{k}+\sum_{i=1}^{K}c_{ai}^{2}b_{ik},\;1\leq k\leq K$$

(10) $$a_{k}=1+\omega_{k}\left\{\left(q_{0k}c_{0k}^{2}-1\right)+\sum_{i=1}^{K}q_{ik}\left[\left(1-p_{ik}\right)+\nu_{i}p_{ik}\rho_{i}^{2}x_{i}\right]\right\}$$

(11) $$b_{ik}=\omega_{k}q_{ik}p_{ik}\nu_{i}\left(1-\rho_{i}^{2}\right)$$

(12) $$x_{i}=1+m_{i}^{-0.5}\left(max\left\{c_{si}^{2},0.2\right\}-1\right)$$

(13) $$\omega_{k}={\left(1+4{\left(1-\rho_{k}\right)}^{2}\left(\gamma_{k}-1\right)\right)}^{-1}$$

(14) $$\gamma_{k}={\left(\sum_{i=0}^{K}q_{ik}^{2}\right)}^{-1}$$

Please note that the first and second order moments for all the internal arrival processes, i.e., the aggregated arrival process to every queuing node in the queuing network, can be computed from the set of linear equations above given the external arrival processes (incoming arrival processes to the queuing system) and the service processes related parameters of the different queues. Table 2 includes the description of the notation considered in the expressions above.

### 4.4. Estimation of the Service Processes Related Input Parameters

Finally, here, we describe methods to estimate the first and second-order moments related to the PRADCs considered in this work. These service times moments are input parameters for both computing the internal arrival processes moments and the per-queue response time described in the previous subsections. Because of the complexity and high domain knowledge required to model some of these input parameters together with their dependency on the scenario specificities (e.g., processor architecture in the second-order moment of the packet processing times), we rely on simulation and experimentation methodologies or combine any of them with mathematical analysis to model many of them. Additionally, we list the factors that most affect them. Please note that the expressions provided next for the service processes related input parameters apply to all the queues modeling the same resource in a given component, even though they refer to different instances.

#### 4.4.1. Packet Transmission at the Transport Network Bridges’ Ouput Ports

Here we consider the packet transmission at the TN bridges’ egress ports as the only PRADC of these components. In this case, the service time is given by the average packet size *L* and the nominal link capacity *C* as L/C. For bridges supporting traffic prioritization as TSN ones, each priority class might have different values of *L*. Regarding the SCV of the packet transmission times, it is mainly given by the packet length distribution, but it is also affected by deviations in the nominal transmission capacity of the link. Experimental measurements can be performed to characterize it.

#### 4.4.2. Packet Processing Times Characterization at the User Plane Function

The primary potential bottleneck of the UPF is related to the higher layers protocols (e.g., GTP-U and PDU) processing. Here, we assume the UPF is deployed as a VNF with one or several dedicated physical CPU cores. The packet processing rate of the UPF per physical CPU core is given by the average number of instructions $I_{UPF}$ to be executed to process a single packet divided by the CPU core power $P_{UPF}$ expressed in instructions per second:

(15) $$\mu_{UPF}=\frac{I_{UPF}}{P_{UPF}}$$

On the other side, the SCV of the UPF’s processing time $c_{s,UPF}^{2}$ is a function of the PM configuration (e.g., CPU governor, C-States, and processor architecture and operation) [26], and the virtualization layer (e.g., Kernel-based Virtual Machine (KVM)). Given the complexity of deriving a model considering all these variables, we rely on experimental measurements of the $c_{s,UPF}^{2}$ in this work.

#### 4.4.3. Packet Processing Times Characterization at the Central Unit

The primary potential bottleneck of the CU is associated with the gNB higher-layers protocols (e.g., SDAP and PDCP) processing. We also assume the CU is deployed as a VNF with one or several dedicated physical CPU cores. Let $P_{CPU}$ and $G_{CU}$ denote the processing power of a CPU core expressed in Giga Operations Per Second (GOPS) and the number of GOPs required to process a single packet in a given gNB-CU instance. Then, the service rate of the gNB-CU instance is given by:

(16) $$\mu_{CU}=\frac{P_{CPU}}{G_{CU}}$$

As the virtualized UPF, the SCV of the virtualized CU $c_{s,CU}^{2}$ will also depend on the PM configuration and the specific virtualization layer. Additionally, it depends on the per UE SINR distribution of the particular scenario. Again, $c_{s,CU}^{2}$ is obtained through experimental measurements.

#### 4.4.4. Packet Processing Times at the Distributed Unit

Based on the model presented in [56], we distinguish two components in the DU processing delay, namely, dynamic processing and remainder user processing. On the one hand, the dynamic processing is related to the user processing, i.e., (de)modulation and (de)coding, which is a linear function of allocated PRBs and MCS [56]. On the other hand, the remainder of user processing includes scrambling, Downlink Control Indicator (DCI) coding, and Physical Downlink Control Channel (PDCCH) coding [56]. Then, we estimate the gNB-DU average packet rate as:

(17) $$\mu_{DU}=\frac{1}{\left(u_{s}+u_{r}\right)\cdot 10^{-6}}$$

where $u_{s}$ and $u_{r}$ are the dynamic processing and remainder user processing components associated with the processing of a single packet, respectively. Several linear fittings of the form $u_{s}\left(N_{PRB},MCS\right)=a_{s}\left(N_{PRB}\right)\cdot MCS+b_{s}\left(N_{PRB}\right)$ to estimate $u_{s}$ are provided in [56] for an Intel-based Sandy Bridge architecture with the CPU frequency set to 3.2 GHz. As observed, $u_{s}$ is a function of the number of PRBs $N_{PRB}$ and the MCS index MCS. For instance, for downlink and $N_{PRB}=25$ PRBs, $a_{s}\left(25\right)=4.9$ and $b_{s}\left(25\right)=24.4$. Regarding $u_{r}$, some measured values are reported in [56] for different virtualization environments and values of $N_{PRB}$. Since these data roughly suggest $u_{r}$ depends linearly on $N_{PRB}$, we estimate $u_{r}$ as $u_{r}=a_{r}\cdot N_{PRB}+b_{r}$, where $a_{r}$ and $b_{r}$ are fitting parameters that depend on the virtualization environment considered (e.g., Linux Container (LXC), Docker or KVM).

#### 4.4.5. Packet Processing Times Characterization at the Radio Unit

The RU packet processing rate $\mu_{RU}$ is related to the processing of the physical layer and depends on the carrier bandwidth and the virtualization layer [54,55,56]. We adopt the base processing model proposed in [56] to estimate $\mu_{RU}$ as below:

(18) $$\mu_{RU}=\frac{1}{\left(C_{RU}+P_{RU}\right)\cdot 10^{-6}}$$

where $C_{RU}$ and $P_{RU}$ are the base offsets for the cell and platform processing, respectively. $C_{RU}$ is a function of the number of PRBs. $P_{RU}$ also depends on the virtualization environment and platform.

Regarding the SCV of the RU processing time, it mainly depends on the computing capacity drift of the PM.

#### 4.4.6. Packet Transmission Times Characterization at the Radio Interface (NR-Uu)

The radio interface is modeled as a GI/D/m queuing system, i.e., general distribution for the arrival process, deterministic service time, *m* servers and infinite room for packets. Then, we can use (4) to estimate the mean response time of the radio interface by considering the SCV of the service time equals zero ($c_{sk}^{2}=0$ in (4)). The number of servers $m_{k}^{\left(rif\right)}$ in the queuing model is estimated as:

(19) $$m_{k}^{\left(rif\right)}=\left\lfloor \frac{N_{PRB}}{E_{b}}\right\rfloor$$

where $N_{PRB}$ is the number of PRBs available at the radio interface and $E_{b}$ is the average number of PRBs required to serve a single packet through the radio interface. The parameter $E_{b}$ can be estimated either experimentally or through simulation as in this work. Either way, observe that this parameter includes the co-channel interference effect. Trivially, the higher the co-channel interference, the greater the number of required PRBs to serve a packet will be.

On the other hand, the service time of the radio interface is given by the chosen numerology, which, in turn, determines the time slot duration τ. Thus, the service rate at the radio interface is given by:

(20) $$\mu_{RIF}=\frac{1}{\tau }$$

QT-based performance models have been proposed and validated in [32,57]. More precisely, the authors in [57] model the radio interface as an M/M/m/K queuing system, i.e., a system with Poissonian arrival and service processes, *m* servers and finite queue length. In [32], the authors propose a more accurate model at the expense of a higher complexity. However, it shall be noted that the accuracy of the M/M/m/K model to estimate the channel Packet Loss Ratio (PLR) is still quite fair according to the results reported in [32] (see [Figure 1.a]). Here, we compute the number of servers as in [57], but we consider deterministic service times as in [32] as we assume there is no HARQ retransmissions. In contrast to [32,57] that consider Poissonian arrival processes, we do not make any assumption on the packet arrival process. In this regard, the model used here is more general.

## 5. Experimental Setup

This section details the scenarios, methods, and configurations considered in this work to carry out our experimentation.

**Figure 3:**
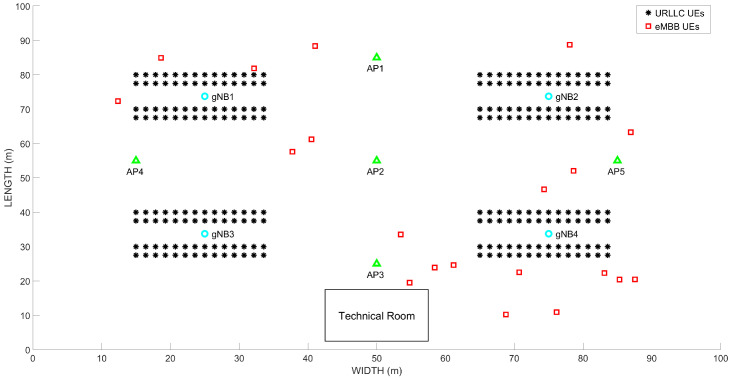
Industrial scenario layout.

Figure 3 shows the scenario employed in our evaluation. More precisely, it includes the layout of the factory floor considered in our setup. As observed, it consists of four PLs, each with fifty-six motion control devices and twenty eMBB users. Moreover, four 5G gNBs and five Wi-Fi Access Points (APs) are part of the industrial scenario RAN. This layout is inspired by the one considered in [69].

**Figure 4:**
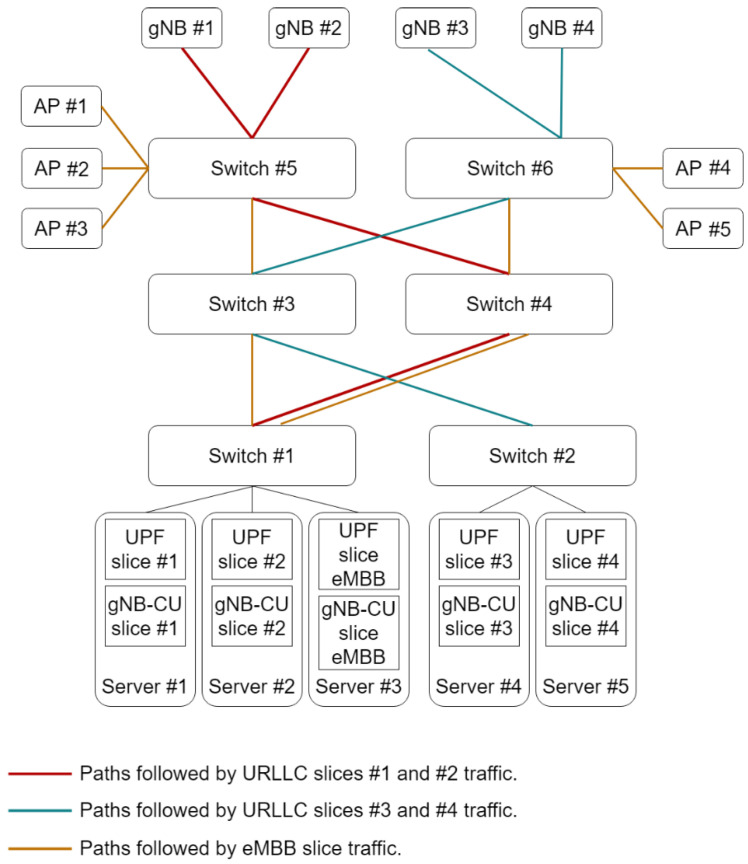
Infrastructure setup for the evaluation.

Figure 4 depicts the underlying network infrastructure together with the placement of the 5G VNFs (e.g., gNB-CU and UPF). The servers and bridges depicted in this figure are physically placed in the technical room shown in Figure 3. The figure also includes the paths followed by each slice in the midhaul network, that is, the TN segment interconnecting the CUs with the gNB-DUs. For the sake of clarity, the path followed by the aggregated traffic from each cluster of servers to a given gNB or AP is specified all along the network. Nonetheless, actually, there is a single full-duplex link interconnecting each bridges pair at most. Then, for instance, the aggregated traffic from URLLC slices #2, #3, and #4 shares the link between TSN switch #6 and TSN switch #7.

We evaluate the E2E mean delay for the following two configurations with the aim of assessing the effectiveness of slicing in terms of isolation:

- Configuration 1: The URLLC traffic generated by each of the four PLs in the factory floor is served by a segregated slice, thus providing isolation between the production lines. The PL #1 generates an aggregated non-conformant traffic that does not meet the aggregated committed data rate due to a failure in its operation.
- Configuration 2: The URLLC traffic generated by all of the four PLs in the factory floor is served by a single slice. The production line #1 generates non-conformant traffic due to a failure in its operation.

We also consider the following two variants for each scenario configuration in order to compare the transport network technologies (e.g., standard (bare) Ethernet, and asynchronous TSN):

- Variant A: The midhaul network in Figure 4 is realized as a standard IEEE 802.1Q Ethernet network where there is no traffic prioritization.
- Variant B: The midhaul network in Figure 4 is implemented as an asynchronous TSN network, whose building block is the ATS. There is an ATS instance at every TSN bridge egress port. The ATS includes a per-flow traffic regulation through the interleaved shaping and traffic prioritization.

The combination of each of the configurations 1 and 2 with the two variants of the TN results in four different scenarios to be evaluated, namely: (i) configuration 1.A (dedicated slice for every PL and standard Ethernet for the midhaul network), (ii) configuration 1.B (dedicated slice for every PL and TSN for the midhaul network), (iii) configuration 2.A (single slice serving the traffic of all the PLs and standard Ethernet for the midhaul network), and (iv) configuration 2.B (single slice serving the traffic of all the PLs and TSN for the midhaul network).

A dedicated 5GS is deployed for each URLLC slice. This 5GS includes dedicated virtualized UPF and gNB-CU instances to serve the traffic generated by the respective production line(s). There are also isolated radio and TN resources destined for the slice. The upper layers of the virtualized UPF and gNB-CU instances follow a FCFS discipline to serve the packets following a RTC strategy. They are instantiated at the edge cluster (placed at the technical room) and have dedicated physical CPU cores for this task (CPU pinning). The gNB-DU and the radio unit are deployed as a small cell (physical network function -PNF-) operating at 3.5 GHz and 100 MHz of bandwidth. For the TN, we consider both standard Ethernet and asynchronous TSN technologies, as commented previously. The constituent TSN bridges of the TSN network include an ATS at every egress port. Every ATS includes eight priority levels and sixteen shaped buffers. The transmission capacity for every link was set to 1 Gbps. Additionally, the PRADCs considered for the downlink at each slice are the UPF upper-layers processing, gNB-CU upper-layers processing, the transmission process at every involved link in the TN, gNB-DU processing, gNB-RU processing, and radio interface transmission process. Considering these bottlenecks, we used the model (3)–(20) described in Section 4 to estimate the E2E mean response time.

**Figure 5:**
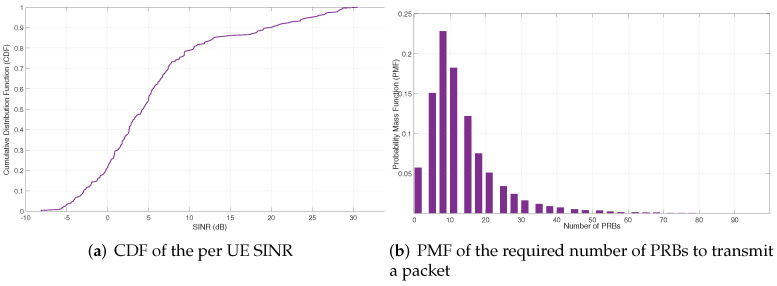
CDF of the SINR and PMF of the number of PRBs obtained through simulations.

The main configuration parameters are included in Table 3. It is worth highlighting that a realistic configuration of the industrial scenario parameters has been taken into consideration. In our setup, we assumed the expected throughput generated by each PL is the same and we performed the dimensioning of the resources for each slice and configuration accordingly. In the same way, each eMBB slice generates the same amount of aggregated traffic for each AP. The quality radio signal-related parameters (e.g., mean number of PRBs required to transmit a URLLC packet at the radio interface ($E_{b}$), average spectral efficiency per user, average SINR per user) in Table 3 were measured through simulation considering the layout shown in Figure 3. Figure 5 includes the Cumulative Distribution Function (CDF) of the per-UE SINR and the Probability Mass Function (PMF) of the PRBs required to transmit a single packet obtained via simulation for the industrial scenario considered.

## 6. Results

This section includes the numerical results obtained from the evaluation of the E2E mean response time for the four configurations presented in the previous section.

**Figure 6:**
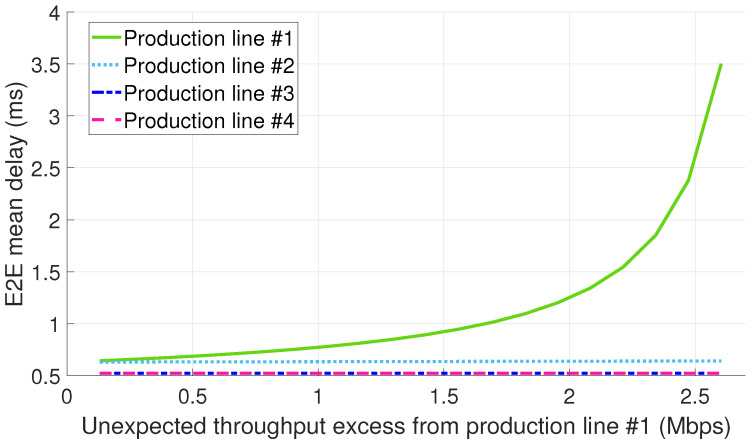
E2E mean delay for configuration 1.A (dedicated slices + std. Ethernet for the midhaul).

Figure 6 depicts the E2E mean packet delay per production line (PL) for the configuration 1.A (see Section 5). The abscissae axis in the figure represents the throughput excess generated by the PL #1 due to a malfunctioning. The results show that only the mean packet delay of the PL #1 is primarily affected by the non-conformant traffic, thus suggesting the effectiveness of infrastructure slicing for ensuring the isolation among slices.

**Figure 7:**
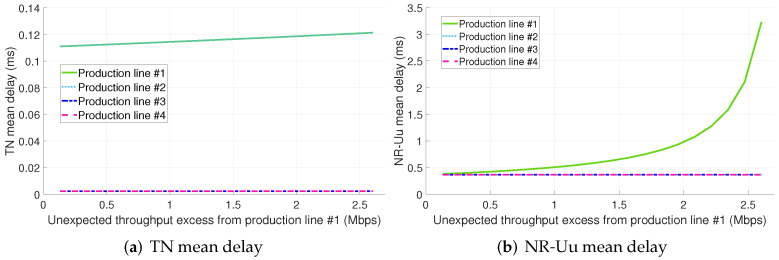
TN and NR-Uu mean delay for configuration 1.A (dedicated slices + std. Ethernet for the midhaul).

Table 4 includes a breakdown of latency per considered PRADC and per studied configuration. Each cell in the table includes the minimum and maximum mean packet delay (expressed in microseconds) obtained per PL in the evaluated range of throughput excess for the respective identified bottleneck and configuration. The entries in the table that include only one value instead of an interval stand for a constant or roughly constant mean delay for all the throughput excess values assessed. As observed, the traffic excess from PL #1 is not impacting the mean packet delay of the 5G components and radio interfaces (i.e., NR-Uu) of the PLs #2, #3, and #4 as their serving slices have dedicated computing and radio resources, respectively. Nonetheless, the non-conformant traffic results in an increase of the TN packet delay for the PL #2. This fact can be clearer observed in Figure 7a. The explanation of this fact is that the standard Ethernet network considered cannot provide per link traffic isolation, i.e., there are no means to reserve a segregated link capacity per slice. Please note that the traffic from PLs #1 and #2 share the same paths in the TN (see Figure 4). Therefore, using bare Ethernet as transport network technology does not ensure the full isolation among slices.

Also, it is remarkable that the TN delay of the slices serving PLs #1 and #2 is significantly higher than the one experienced by the traffic from PLs #3 and #4. That is due to the fact that slices #1 and #2 are sharing the link from switch #1 to switch #4 with eMBB traffic (see Figure 4) and there is no traffic prioritization.

The main bottleneck in configuration 1.A is the radio interface (see Figure 7b). Please note that, even considering there are dedicated radio resources for each PL, in a real scenario it is expected to observe an increase in the mean packet delay at the radio interface of the slices serving PLs #2, #3 and #4 with the PL #1 traffic load excess. This is because of the interference and it depends on the per gNB radio resources to slices assignment.

**Figure 8:**
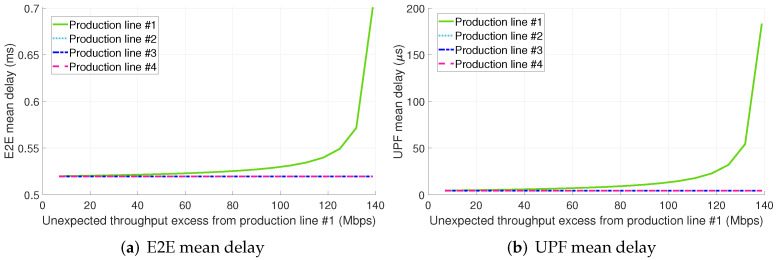
E2E and UPF mean delay for configuration 1.B (dedicated slices + TSN for the midhaul).

Figure 8a shows the E2E mean packet delay per PL for the configuration 1.B. In contrast to configuration 1.A, in this configuration TSN is used as L2 technology in the TN. In this case, the results also suggest that the traffic excess from PL #1 does not have any impact on the rest of PLs. It is noteworthy that this configuration requires a higher throughput excess to significantly degrade the performance perceived by PL #1 traffic. This is related to the fact that the asynchronous TSN TN performs a per flow traffic regulation at every TSN bridge egress port, thus filtering the non-conformant traffic. As a consequence, the UPF becomes the main bottleneck of the network as shown in Figure 8b. For the same reason, the mean packet delays of the TN, gNB-DU, gNB-RU, and radio interface for PL #1 do not depend on the traffic excess. Last, please note that the traffic from PLs #1 and #2 experiences the longest TN delays (see Table 4). Although the asynchronous TSN TN includes traffic prioritization, the transmissions are non-preemptive and therefore the eMBB traffic still degrades the performance of PLs #1 and #2 traffic in the link interconnecting switches #1 and #4 (see Figure 4).

**Figure 9:**
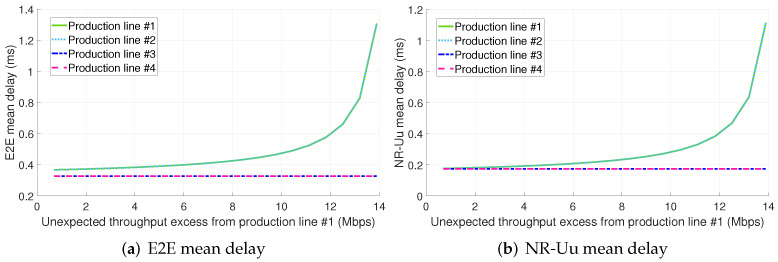
E2E and NR-Uu mean delay for configuration #2.A (shared slice + std. Ethernet for the midhaul).

Figure 9a depicts the E2E mean packet delay for configuration 2.A. In contrast to configurations 1.A and 1.B, the non-conformant traffic from the PL #1 severely degrades the performance perceived by the traffic from PL #2. This fact further highlights the effectiveness of infrastructure slicing for providing isolation. On the other hand, the mean packet delay of PLs #3 and #4 seems to remain independent of the PL #1 traffic excess despite there is a single slice to serve the traffic from all the PLs. As in configuration 1.A, the primary bottleneck of configuration 2.A is the radio interface as shown in Figure 9b. The traffic from PL #1 is only sharing the computational and radio resources at the RAN with PL #2. Consequently, the traffic from PLs #3 and #4 only perceives an increase in its delay at the UPF and gNB-CU. However, the utilization of the UPF and gNB-CU resources is low compared to the radio ones and the performance degradation experienced by PLs #3 and #4 traffic is negligible in the range of throughput excess studied.

**Figure 10:**
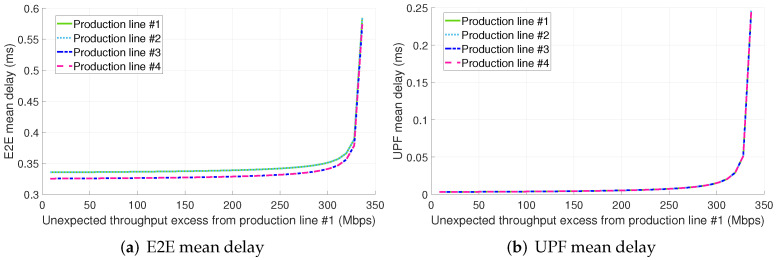
E2E and UPF mean delay for configuration 2.B (shared slice + TSN for the midhaul).

Finally, in contrast to the previous configurations, the traffic excess from PL #1 significantly increases the E2E mean packet delay of all the PLs in configuration 2.B (see Figure 10a).

As in configuration 1.B, the UPF becomes the primary bottleneck since the TSN TN does not allow the traffic excess pass through (see Figure 10b). Notably, it is apparent, especially for low values of the throughput excess, that the E2E mean packet delay is slightly higher for PLs #1 and #2. This is because of non-preemptive transmissions of the eMBB traffic at the TSN TN as explained for configuration 1.B. Also, it shall be noted that the throughput excess to overload the bottleneck is greater than in the configurations previously discussed due to the two following reasons:

(i) Compared to configurations 1.A and 2.A, the bottleneck in this configuration is the UPF, which has an initial utilization much lower than radio resources given our setup.
(ii) The throughput excess in this configuration leverages statistical multiplexing to utilize the UPF computational resources surplus allocated to PLs #2, #3, and #4 in configuration 1.B.

## 7. Conclusions and Future Work

In this article, we have proposed a queuing theory-based model to estimate the end-to-end mean delay of 5G infrastructure network slices. Then, using this model, we have investigated the effectiveness of infrastructure in terms of the degree of isolation in industrial private 5G networks. To that end, we have considered a reasonably complete and realistic setup whose main parameters have been obtained from experimentation and simulation. The use case addressed in this work aims to show the benefits brought by using segregated infrastructure slices, each with dedicated resources at every network domain, for serving the traffic generated by the different PLs in the factory floor. In this way, we might reduce the number of production downtimes and the corresponding associated expenditures. This is because the traffic excess from any PL due to any malfunctioning will not negatively affect the operation of the rest of PLs as a consequence of a QoS degradation in the network.

As concluding remarks, our results suggest the effectiveness of infrastructure network slicing in ensuring a quite fair degree of isolation among segregated slices. Nonetheless, the use of standard (bare) Ethernet does not ensure the complete isolation of the slices as it does not include support for traffic prioritization and resources reservation. In this way, for example, the eMBB cross traffic at the different links of the TN interferes and degrades the performance of URLLC services. This might potentially result in production downtimes. To overcome this issue, TSN technology might be used to enable a per link dedicated resources assignment to every slice. Furthermore, TSN enhances the performance of the TN segments due to its traffic prioritization capability, thus drastically reducing the adverse effects of the interfering eMBB traffic.

As future work, several challenges lie ahead. One of the central challenges is to devise, develop and validate a solution for automating the management and operation of the industrial infrastructure slices. The principal objective might be to minimize the expenditures associated with the production downtimes while using the minimum resources necessary to ensure the target QoS metrics for the proper operation of the involved industrial services. Solving this challenge requires further research to address many currently open issues. First, it is necessary to integrate the knowledge from OT, Information Technology (IT), and economic domains to model the production downtimes cost as a function of the network performance metrics. Then, the resulting model could drive the solution towards the aforementioned goal. Second, network calculus-based models for the delay and jitter (delay variation) must be developed to holistically capture the network slices’ behavior. Existing related works only capture network parts of the features and operation of the slice. These models are needed for a feasibility check of the configurations issued by the solution, i.e., to verify whether a given configuration meets the delay and jitter requirements of the involved industrial services. Similarly, stochastic models are a must to estimate the network slice availability. Then, the solution could harness them to compute, for instance, the required redundancy to ensure the availability requirements. Last, likely, the use of Machine Learning (ML) techniques is required to assist the optimization process in coping with its complexity.

The challenge of realizing a zero-touch solution for managing industrial network slices is accentuated in industrial networks that integrate 5G and TSN. The integration of 5G with TSN, which 3GPP is addressing (see 3GPP TS 23.501), is crucial to realize tomorrow’s converged industrial networks, providing both wired and wireless access with deterministic QoS support. In this way, these networks will satisfy the needs of almost any industrial service. In this scenario, the 5GS is regarded as a set of virtual TSN bridges that can be logically configured by the TSN controller through the TSN application function at the 5G control plane. This scenario brings further problems. For example, the coordination and cohesion of the configurations of 5G and TSN segments must be ensured. On the one hand, the delay and jitter budgets of the industrial services have to be properly distributed between these two segments. On the other hand, the deterministic QoS requirements and configurations issued by the TSN controller for the 5G virtual TSN bridges have to be translated into a valid setup for the 5GS.

## Figures and Tables

**Table 1:** Related literature addressing the analytical modeling of the network slices’ E2E delay.

References	Mathematical Framework			Description
	QT	DNC	SNC	
Schulz et al. [16]	✗			This work aims to provide an E2E delay model for a mobile network. To that end, it proposes a model to estimate the sojourn time distribution of the GI/GI/1 queue and assumes Kleinrock’s independence approximation. The model is tested and validated for a single M/D/1 queue with different scheduling policies.
Ye et al. [25]	✗			This article models the E2E delay traversing a VNF chain. The primary assumption considered is the system bottlenecks are the CPU processing and link transmission, both following a generalized processor sharing discipline for service. The proposed model consists of an independent tandem of M/D/1 queues for each flow.
Xu et al. [17,18]			✗	This paper derives statistical E2E delay bounds for network slicing considering Gaussian traffic and deterministic service. This model is leveraged to perform dynamic resource provisioning, i.e., to adjust the slice allocated resources according to the traffic fluctuations. Specifically, resource dimensioning is carried out using the derived performance bounds.
Yu et al. [19]			✗	This article provides stochastic performance bounds for network slices using martingale-based approaches. The resulting bounds are employed to translate delay requirements into bandwidth ones and to estimate the power allocation at the RAN considering an ALOHA-like medium access technique for URLLC traffic.
Sweidan et al. [20]	✗			This work studies the joint problem of E2E networks slices composition, the mapping of URLLC applications to slices, and multiple disjoint paths to slices assignment. It models the E2E mean delay of a network slice as an open network of M/M/1 queues.
Fantacci et al. [21]			✗	This article relies on martingale theory to derive statistical bounds of the slices E2E packet delay. The model is applied for virtual network embedding. It focuses on ultimate VR services operated in 6G Terahertz networks. They validate the bounds through simulation and compare their accuracy with an equivalent Markov tandem queue model.
Liu et al. [23]		✗		This paper presents a worst-case delay model for virtual wireless networks, including physical and virtual nodes. It considers that different slices might receive differentiated treatment through the use of virtual queues.
Picano [22]			✗	This work aims to evaluate the performance of the Sixth Generation (6G) pervasive edge computing network for handling virtual reality traffic for two scheduling policies, namely, First Come, First Served (FCFS) and earliest deadline first. To that end, it relies on a martingale-based model similar to the one proposed in [21].
Chien et al. [24]	✗			This article proposes a solution for slices capacity allocation and traffic offloading from the central office to the edge cloud. The solution relies on an E2E mean delay model consisting of a feedforward network of M/M/1 queues, each standing for either a node or a link. The solution is validated experimentally.
Kalør et al. [27]		✗		This paper focuses on modeling the E2E delay of URLLC network slices using DNC for deterministic and switched networks. It presents an industrial medicine manufacturing system as a case study to illustrate the usefulness of DNC for analyzing the worst-case E2E delay of network slices.

**Table 2:** Main notation.

Notation	Description
Variables related to the E2E mean response time computation	
*K*	Number of queues in the queuing network.
Φ	Constant delays in the system.
*T*	Mean response time of a network slice.
$T_{k}$	Mean sojourn time at queue *k*.
$V_{k}$	Visit ratio of queue *k*.
$\lambda_{0k}$	Mean external arrival rate at queue *k*.
$c_{0k}^{2}$	SCV of the external arrival process at queue *k*.
$m_{k}$	Number of servers at queue *k*.
$c_{ak}^{2}$	SCV of the inter-arrival packet times at queue *k*.
$\mu_{k}$	Average service rate at queue *k*.
$\mu_{k}^{\left(p\right)}$	Average service rate at queue *k* for priority class *p*.
$c_{sk}^{2}$	SCV of the service time at queue *k*.
$c_{sk}^{2(r)}$	SCV of the service time at queue *k* for priority class *p*.
$p_{ik}$	Probability that a packet leaves node *i* to node *k*.
$\nu_{i}$	Multiplicative factor for the flow leaving queue *i*.
$d_{ik}$	Link delay between queues *i* and *k*.
C(m,ρ)	The Erlang’s C formula.
$a_{k}$, $b_{ik}$	System of equations coefficients for computing the mean and squared coefficient of variation (SCV) of the inter-arrival packet times to every queue.
$\omega_{k}$, $x_{i}$, $\gamma_{k}$	Auxiliary variables for $a_{k}$ and $b_{ik}$ computation.
$q_{0k}$	Proportion of arrivals to node *k* from its external arrival process.
$q_{ik}$	Proportion of arrivals from node *i* to node *k*.
$\rho_{k}$	Link utilization at queue *k*.
$\rho_{k}^{\left(p\right)}$	Link utilization for queue *k* for priority class *p*.
$T_{NPMP-MG1}^{\left(p\right)}$	Mean delay of a non-preemptive multi-priority queue for priority class *p*.
$T_{GGm}$	Mean delay estimation of a G/G/m queue.
$\lambda_{k}$	Aggregated arrival rate at queue *k*.
$\lambda_{k}^{\left(p\right)}$	Aggregated mean packet arrival rate of queue *k* for priority class *p*.
Variables of service processes related input parameters	
*L*	Average packet size.
*C*	Nominal link capacity.
$\mu_{UPF}$	UPF packet processing rate per physical CPU core.
$I_{UPF}$	Number of instructions to be executed to process a single packet.
$P_{UPF}$	CPU power.
$\mu_{CU}$	gNB-CU serving rate.
$P_{CPU}$	CPU power.
$G_{CPU}$	Number of Giga OPerationss (GOPs) required to process a single packet in a given gNB-CU instance.
$\mu_{DU}$	gNB-DU average packet rate.
$u_{s}$	Dynamic processing component.
$u_{r}$	Remainder user processing component.
$\mu_{RU}$	RU packet processing rate.
$C_{RU}$	Base offset for the cell processing.
$P_{RU}$	Base offset for the platform processing.
$m_{k}^{\left(rif\right)}$	Number of servers in the radio interface.
$N_{PRB}$	Number of PRBs available at the radio interface.
$E_{b}$	Average number of PRBs required to serve a single packet.
$\mu_{RIF}$	Service rate at the radio interface.
τ	Time slot duration.

**Table 3:** Main configuration parameters.

Parameters	Value
Number of production lines	4
Number of URLLC flows per production line	56
URLLC service	Motion Control (MC) [70,71]
Packet delay budget MC	1 ms [70,71]
Packet length MC	80 bytes
Sustainable rate per MC flow	1.55 Mbps [70,71]
Burstiness per MC flow	2592 bits
eMBB traffic generated from server #3 to each Wi-Fi AP	AP#1: 330 Mbps, AP#2: 330 Mbps, AP#3: 330 Mbps AP#4: 800 Mbps, AP#5: 800 Mbps
eMBB packet size	1500 bytes
UPF service rate per processing unit (CPU core)	357,140 packets per second (from data included in [33])
SCV of the UPF service time	0.65 (from experimental measurements in [26])
gNB-CU service rate per processing unit (CPU core)	601,340 packets per second (from data included in [55])
SCV of the gNB-CU service time	0.65 (from experimental measurements in [26])
CPU core power (Intel Xeon Platinum 8180)	25.657 GOPS
gNB-DU service rate per processing unit (CPU core)	Substitute $a_{s}=0.097\cdot E_{b}+2$, $b_{s}=1.6\cdot E_{b}-14$, and $u_{r}=1.3\cdot E_{b}+23$ in (17) (fittings derived from experimental data in [56])
SCV of the gNB-DU service time	1
gNB-RU service rate per processing unit	Substitute $C_{RU}=1.2\cdot E_{b}-11$, and $P_{RU}=0.03\cdot E_{b}+4.3$ in (18) (fittings derived from experimental data in [56])
SCV of the gNB-RU service time	1
Processing units allocated to each network component. The number of processing units were designed to ensure that the utilization of the computing resources for every component is lower than 75%.	Configuration 1: UPF: 1 CPU core (Intel Xeon 8081) gNB-CU: 1 CPU core (Intel Xeon 8081) gNB-DU: 24 CPU cores (Intel SandyBridge i7-3930K @3.20Ghz gNB-RU: 3 CPU cores (Intel SandyBridge i7-3930K @3.20Ghz) Configuration 2: UPF: 3 CPU cores (Intel Xeon 8081) gNB-CU: 2 CPU cores (Intel Xeon 8081) gNB-DU: 96 CPU cores (Intel SandyBridge i7-3930K @3.20Ghz) gNB-RU: 10 CPU cores (Intel SandyBridge i7-3930K @3.20Ghz)
Visit ratios of the UPF and gNB-CU	1
Visit ratios of the gNB-DU, gNB-RU and radio interface	0.5
TSN links capacities	All links have a capacity of 1 Gbps
MC traffic-to-priority level assignment at every TSN bridge output port	1 (1 is the highest priority level and 8 is the lowest)
eMBB traffic-to-priority level assignment at every TSN bridge output port	8
PRB bandwidth	180 kHz
Radio interface time slot duration	142.8 μs
Number of PRBs dedicated for each URLLC slice per gNB	Configuration 1: Slice#1: gNB#1: 166, gNB#2: 166, gNB#3: 0, gNB#4: 0 Slice#2: gNB#1: 166, gNB#2: 166, gNB#3: 0, gNB#4: 0 Slice#3: gNB#1: 0, gNB#2: 0, gNB#3: 166, gNB#4: 166 Slice#4: gNB#1: 0, gNB#2: 0, gNB#3: 166, gNB#4: 166 Configuration 2: Slice#1: gNB#1: 333, gNB#2: 333, gNB#3: 333, gNB#4: 333
Mean number of PRBs required to transmit a URLLC packet at the radio interface ($E_{b}$)	15.8
Average spectral efficiency per user	2.8173 bps/Hz (MCS index=22)
Average SINR per user	3.5368 dB
External arrival process (to the UPF)	Poissonian

**Table 4:** Mean delay (expressed in microseconds) contribution per component and per production line.

	ConFigure 1.A	ConFigure 1.B	ConFigure 2.A	ConFigure 2.B
TN	PL#1-2: 111.10-121.40 PL#3-4: 2.25	PL#1-2: 40.16-41.87 PL#3-4: 2.07	PL#1-2: 40.16-41.87 PL#3-4: 2.07	PL#1-2: 12.18 PL#3-4: 2.07
UPF	PL#1: 4.22-4.28 PL#2-4: 4.21	PL#1: 4.41-183.20 PL#2-4: 4.21	PL#1-4: 3.18-3.23	PL#1-4: 3.21
CU	PL#1: 2.09-2.10 PL#2-4: 2.09	PL#1: 2.13-4.36 PL#2-4: 2.09	PL#1-4: 2.04-2.08	PL#1-4: 2.06-7.94
DU	PL#1: 13.25 PL#2-4: 13.25	PL#1-4: 132.50	PL#1-4: 132.50	PL#1-4: 132.50
RU	PL#1:1-2: 13.13-13-16 PL#2-4: 13.13	PL#1-4: 13.13	PL#1-2: 12.88-12.93 PL#3-4: 12.88	PL#1-4: 12.87
NR-Uu	PL#1: 379.80-3229.00 PL#2-4: 367.60	PL#1-4: 367.60	PL#1-2: 174.20-1114.00 PL#3-4: 174.20	PL#1-4: 172.60

## Data Availability

Not applicable.

## Abbreviations

The following abbreviations are used in this manuscript:

**Acronym**	**Acronym expansion**
3GPP	3rd Generation Partnership Project
4G	Fourth Generation
5G	Fifth Generation
5GS	5G System
AGV	Automated Guided Vehicle
AP	Access Point
AR	Augmented Reality
ATS	Asynchronous Traffic Shaper
BBU	Baseband Unit
BE	best-effort
CBS	Credit-Based Shaper
CDF	Cumulative Distribution Function
CN	Core Network
CPU	Central Processing Unit
CU	Central Unit
DCI	Downlink Control Indicator
DNC	Deterministic Network Calculus
DU	Distributed Unit
DRP	Dynamic Resource Provisioning
E2E	end-to-end
eMBB	enhanced Mobile Broadband
EPC	Evolved Packet Core
FCFS	First Come, First Served
gNB	Next Generation NodeB
GOP	Giga OPerations
GOPS	Giga Operations Per Second
HARQ	Hybrid Automatic Repeat Request
IFFT	Inverse Fast Fourier Transform
IT	Information Technology
KVM	Kernel-based Virtual Machine
L2	layer 2
LXC	Linux Container
LTE	Long-Term Evolution
MAC	Medium Access Control
MCS	Modulation and Coding Scheme
MIMO	Multiple-Input Multiple-Output
ML	Machine Learning
MTBF	Mean Time Between Failures
NF	Network Function
NFV	Network Functions Virtualisation
NP	nondeterministic polynomial time
NR	New Radio
NS	Network Softwarization
OP	Operational Technology
PCP	Priority Code Point
PDCP	Packet Data Convergence Protocol
PL	production line
PLR	Packet Loss Ratio
PMF	Probability Mass Function
PM	Physical Machine
PRADC	Primary Resource Access Delay Contribution
PRB	Physical Resource Block
QoS	Quality of Service
QN	Queuing Network
QNA	Queuing Network Analyzer
QT	Queuing Theory
RAM	Random Access Memory
RAN	Radio Access Network
RF	Radio Frequency
RLC	Radio Link Control
RRC	Radio Resource Control
RTC	run-to-completion
RU	Radio Unit
SCV	squared coefficient of variation
SDN	Software-Defined Networking
SDAP	Service Data Adaptation Protocol
SINR	Signal-to-Interference-plus-Noise Ratio
SNC	Stochastic Network Calculus
SNS	Softwarized Network Service
TAS	Time-Aware Shaper
TN	Transport Network
TR	Technical Report
TSN	Time-Sensitive Networking
UE	User Equipment
URLLC	Ultra-Reliable and Low Latency Communication
UP	User Plane
UPF	User Plane Function
VLAN	Virtual Local Area Network
VNF	Virtual Network Function
VNFC	Virtual Network Function Component
VR	Virtual Reality
WAT	Wireless Access Technology
